# art.pics Database: An Open Access Database for Art Stimuli for Experimental Research

**DOI:** 10.3389/fpsyg.2020.576580

**Published:** 2020-12-16

**Authors:** Ronja Thieleking, Evelyn Medawar, Leonie Disch, A. Veronica Witte

**Affiliations:** ^1^Department of Neurology, Max Planck Institute for Human Cognitive and Brain Sciences, Leipzig, Germany; ^2^Berlin School of Mind and Brain, Humboldt-Universität zu Berlin, Berlin, Germany; ^3^Charité – Universitätsmedizin Berlin, Humboldt-Universität zu Berlin, Berlin, Germany

**Keywords:** art, subjective value, stimuli, fMRI, reward, remuneration, experimental database, wanting and liking

## Abstract

While art is omnipresent in human history, the neural mechanisms of how we perceive, value and differentiate art has only begun to be explored. Functional magnetic resonance imaging (fMRI) studies suggested that art acts as secondary reward, involving brain activity in the ventral striatum and prefrontal cortices similar to primary rewards such as food. However, potential similarities or unique characteristics of art-related neuroscience (or neuroesthetics) remain elusive, also because of a lack of adequate experimental tools: the available collections of art stimuli often lack standard image definitions and normative ratings. Therefore, we here provide a large set of well-characterized, novel art images for use as visual stimuli in psychological and neuroimaging research. The stimuli were created using a deep learning algorithm that applied different styles of popular paintings (based on artists such as Klimt or Hundertwasser) on ordinary animal, plant and object images which were drawn from established visual stimuli databases. The novel stimuli represent mundane items with artistic properties with proposed reduced dimensionality and complexity compared to paintings. In total, 2,332 novel stimuli are available open access as “art.pics” database at https://osf.io/BTWNQ/ with standard image characteristics that are comparable to other common visual stimuli material in terms of size, variable color distribution, complexity, intensity and valence, measured by image software analysis and by ratings derived from a human experimental validation study [*n* = 1,296 (684f), age 30.2 ± 8.8 y.o.]. The experimental validation study further showed that the art.pics elicit a broad and significantly different variation in subjective value ratings (i.e., liking and wanting) as well as in recognizability, arousal and valence across different art styles and categories. Researchers are encouraged to study the perception, processing and valuation of art images based on the art.pics database which also enables real reward remuneration of the rated stimuli (as art prints) and a direct comparison to other rewards from e.g., food or money.

**Key Messages:** We provide an open access, validated and large set of novel stimuli (*n* = 2,332) of standardized art images including normative rating data to be used for experimental research. Reward remuneration in experimental settings can be easily implemented for the art.pics by e.g., handing out the stimuli to the participants (as print on premium paper or in a digital format), as done in the presented validation task. Experimental validation showed that the art.pics’ images elicit a broad and significantly different variation in subjective value ratings (i.e., liking, wanting) across different art styles and categories, while size, color and complexity characteristics remained comparable to other visual stimuli databases.

## Introduction

Human behavior relies on subjective values, emerging from physiological and cultural needs such as food, money and art. However, the underlying neurobiology of how we perceive, process and differentiate a certain value that is elicited by an external stimulus is far from fully understood, especially with regard to art (Chatterjee, 2011). Notably, rewards can be hierarchically clustered into primary (water, food, and sex) and secondary rewards (money, social cues, esthetics, and engaging activities), with potentially different processing signatures in the brain.

While primary rewards are evolutionary imprints and hardly learnable, humans learn throughout life to derive pleasure from secondary rewards such as art (for a detailed discussion, see *The Esthetic Brain* by Chatterjee, 2014). The subjective value of a certain stimulus is also reflected by its “liking” and/or “wanting,” two different concepts that likely involve different brain mechanisms. While liking often refers to the “actual pleasurable impact of reward consumption,” wanting refers to a form of motivation, or incentive salience of a given stimulus (Berridge and Robinson, 2016). For esthetic pleasure there seem to be distinct characteristics compared to appetitive pleasures: esthetics commonly rely on complex mechanisms that are associated with a mixture of emotions unlike appetitive liking, and, as art is not an instinct, esthetics highly depends on individual experience and knowledge based on esthetic encounters (Chatterjee, 2014).

Although old in human history, the understanding of art gained a new momentum with the emerging research field of neuroesthetics that aims to develop a better understanding of the perception, production and response to art, including neural measures. Moreover, recent research proposes effects of art that go beyond only “pleasure” but also toward a beneficial effect on our psychological well-being (Christensen and Gomila, 2018). However, using art stimuli as universally pleasant stimuli is a naïve endeavor, because it has been shown that liking ratings in experimental settings are highly dependent on the context and the viewer (reviewed in Chatterjee and Vartanian, 2016). A study investigating the impact of whether abstract paintings are classified as art objects or not on liking showed that 75% of the stimuli were not considered to be art, however, liking ratings for considered art stimuli was 20% higher (Pelowski et al., 2017).

Using task-dependent functional magnetic resonance imaging (fMRI), studies showed blood oxygen-level dependent (BOLD)-related brain activity in the ventral striatum (vSTR) and ventromedial prefrontal cortex (vmPFC), areas of the reward-network, in response to expected rewards or penalties (Schultz et al., 1997; Bartra et al., 2013). In addition, further prefrontal areas such as the orbito – and dorsolateral prefrontal cortex (OFC, dlPFC) are discussed to exert top-down control of impulses and emotions that modulate the subjective value of a given object (Hutcherson et al., 2012; Schmidt et al., 2018). Here, liking and wanting are supposedly encoded in distinct neural systems: liking (i.e., hedonic impact) has been predominantly linked to mu-opioid and cannabinoid receptor-related signaling, whereas wanting (i.e., incentive salience) rather relates to dopaminergic signaling (Berridge et al., 2009). Both systems involve the ventral striatum, however, map onto different subparts of the nucleus accumbens (Berridge and Robinson, 2016).

Visual and psychological processes related to art perception and processing have been proposed previously (Ramachandran and Hirstein, 1999; Leder et al., 2004), and neuroscientific studies assessed and localized brain activity in relation to esthetic value (Cela-Conde et al., 2004; Kawabata and Zeki, 2004; Vartanian and Skov, 2014; Lebreton et al., 2015). While esthetic value is considerably subjective, a recent study shows that (visual) esthetic value can be predicted by brain activity based on the integration and different weighing of (visual) features of the presented art image (Iigaya et al., 2020), including low-level (hue, saturation, lightness, color, brightness, blurring effects, edge detecting) (Li and Chen, 2009) and high-level features (color temperature, depth, abstract, emotion, complexity) (Chatterjee et al., 2010). Thus, presumably, primary and secondary rewards are not “randomly” processed in the brain but have – at least to a certain extent – a common ground in human brain computations of stimulus features, which have most likely evolved to serve adaptive behaviors in different environments (Skov and Nadal, 2018; Skov and Skov, 2019).

How a reward’s subjective value is constructed in the brain has been studied e.g., with regard to food: subjective preference for food items is linearly correlated with brain activity in the OFC by the respective macronutrient contents, such as sugar and fat (Suzuki et al., 2017). Others showed that fat and carbohydrate content elicit a supra-additive response for food valuation in the ventral striatum independent of liking (DiFeliceantonio et al., 2018), further highlighting that the brain’s reward evaluation for food involve nutrient sensors in the gut (De Araujo et al., 2020). Considering art evaluation, a recent preprint suggests that feature integration of artistic stimuli might be ordered in an hierarchical way from visual processing up to the integration from low- and high-level image features in the brain, in particular in higher-order areas such as parietal and prefrontal cortex (Iigaya et al., 2020). While it might seem counterintuitive to want art similar to wanting food, it has been argued that art objects, such as prints of art paintings or photographs, are often object of desire, not only of art collectors (Berridge and Kringelbach, 2008).

Convergent brain areas encoding subjective value representation irrespective of reward type are the vSTR and the vmPFC – however, only the vmPFC seems to represent rewards on a common scale in a domain-general manner (Levy and Glimcher, 2011; Gross et al., 2014). A recent meta-analysis further points to a general representation of value as one function of the vmPFC showing convergent activity for both beautiful visual art and beautiful faces (Hu et al., 2017). Thus, art as a secondary reward may elicit the same value-related brain activity patterns in the vmPFC compared to primary rewards such as food, proposing a common, higher-order representation of subjective value.

However, knowledge about (secondary) reward-related neurobiology is still fragmentary, especially with regard to art and related value representations. The encoding of art viewing and experiencing seems to be multi-fold and research on its neural correlates has only begun to discover specific brain signaling (Chatterjee and Vartanian, 2016; Iigaya et al., 2020). Viewing artworks, i.e., paintings, elicits for example activation of the default mode network (DMN) and in subcortical areas like the striatum in relation to the ratings of the painting (Vessel et al., 2012, 2019). The DMN activity seems to be dynamically time-locked to the dynamics of on- and offset of art stimuli, at least for liked ones (Belfi et al., 2019). However, the functional meaning of this remains unclear, also partly due to a lack of adequate experimental tools.

In tasks used for fMRI and other controlled psychological testings, a huge number of repetitions with well-balanced image characteristics of the presented stimuli are essential to generate reliable results (Neseliler et al., 2017). Although a handful of well-documented and widely used food and object image databases are available for such tasks, i.e., food-pics (Blechert et al., 2019), the FoodCast research image database (FRIDa) (Foroni et al., 2013), Full4Health (Charbonnier et al., 2016), those for art pictures are somewhat less comprehensive and mostly contain selected original artworks or stock photographs, like the Catalogue of Art Museum Images Online database (CAMIO)^1^ or the Esthetic Pictures of Everyday Design Products (ADEP) (Yeh et al., 2015) or the Open Affective Standardized Image Set OASIS database (Kurdi et al., 2017). Indeed, the OASIS database provides standardized images along with ratings of valence, arousal and beauty (Kurdi et al., 2017; Brielmann and Pelli, 2019), yet the stimuli are too complex to directly compare to other reward stimuli (such as food) in terms of visual size, complexity and color. To enable for example fMRI assessment of art compared to other rewards, image characteristics have to be carefully matched. In addition, for longitudinal experimental designs a large database of stimuli is needed to control for set image characteristics and to ensure novelty of the presented stimuli for the participant during testing, yet such a database is so far lacking. Therefore, we aimed to create a novel open access database to allow future studies to choose from a well-documented, validated in terms of liking, wanting, recognizability, arousal and valence, and profoundly large set of stimuli (*n* = 2,332) of novel art images (termed *art.pics* from now on) to be used for experimental research.

## Materials and Methods

### Stimuli

To design a large art image dataset, we transformed ordinary visual stimuli (i.e., images) from several databases into standard art stimuli. Therefore, pictures were taken from two large food-related image databases, namely the food-pics_extended database (Blechert et al., 2019) and the FoodCast research image database (FRIDa) (Foroni et al., 2013), adding up to a total of 2,088 images. Original images were provided on the basis of a license agreement with the authors and are available at their respective online resources for food-pics^2^ and FRIDa^3^.

Out of these two databases, we de-selected all food images and selected pictures based on the categories “animals,” “objects” and “plants,” resulting in a set of 728 pictures, 315 from food-pics, and 413 from FRIDa. Importantly, object pictures were only selected if the content was not food-related in order to serve as contrast to other reward stimuli, i.e., food pictures. Further, pictures containing objects with registered labels were excluded due to possible confounding of the brand awareness.

**FIGURE 1:**
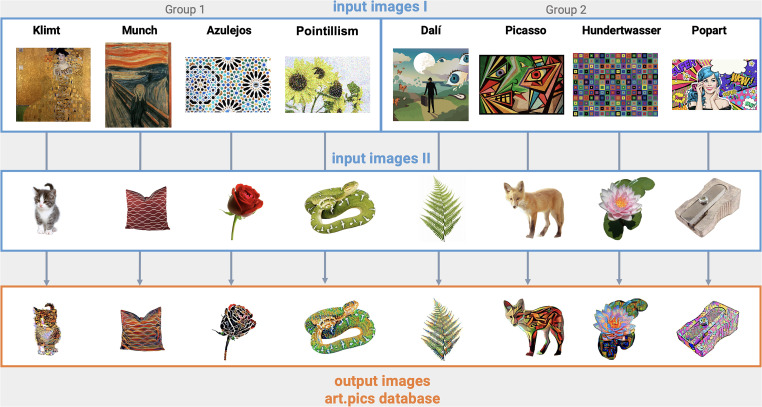
Work flow of the art.pics creation. Input images I (eight different paintings and illustrations) and input images II [animal, object and plant images from two databases: food-pics (Blechert et al., 2019) and FRIDa (Foroni et al., 2013)] are “repainted” by a convolutional neural network (CNN) with art styles learned from the input images I to create images for the art.pics database. Note that original input images for Pointillism, Dalí and Hundertwasser are different to the ones displayed here. Image rights: Klimt “Gustav Klimt, Portrait of Adele Bloch-Bauer, 1907” (Wikimedia Commons), Munch “Edvard Munch, The Scream, 1893” (Wikimedia Commons), Azulejos (Dreamstime), Pointillism (Shutterstock), Dalí (Shutterstock), Picasso (Instagram@rod.harrell), Hundertwasser (Shutterstock), and Popart (Dreamstime).

To obtain art.pics out of this pre-selected dataset, we transformed the initial images into art pictures reflecting eight popular art styles by applying a deep learning algorithm [described in the section “Deep Learning Algorithm (Art Filter)”]. Paintings and illustrations were selected to cover sufficiently distinct styles, including a spectrum of different colors and shapes that would be applied on the original pictures. Detailed description of the art styles is found in Table 1 and original paintings and illustrations are found in Figure 1.

**TABLE 1:** Selected art styles including source and assigned group for creation of art.pics.

Group	Style name	Based on.
1	Azulejos	Tile pattern found in the Alhambra, granada, Spain
1	Klimt	Gustav Klimt, Portrait of Adele Bloch-Bauer, 1907
1	Munch	Edvard Munch, The Scream, 1893
1	Pointillism	Georges Seurat
2	Dalí	Salvador Dalí. The Ship with Butterfly Sails, 1937
2	Hundertwasser	Hundertwasser, The Windows are going Home, 1979
2	Picasso	Cubism style based on Pablo Picasso
2	Popart	Illustration of Blue Head Girl on Pop Art Background

Original pictures and art styles based on famous European artists were divided randomly in two groups and assigned to each other; this way each original picture is represented in four different art styles. Group 1 contains 44 animals, 213 objects and 34 plants which are therefore available in the styles “Azulejos,” “Klimt,” “Munch,” and “Pointillism”; Group 2 contains 46 animals, 220 objects and 31 plants and are therefore available in the styles “Dalí,” “Hundertwasser,” “Picasso,” and “Popart.”

Art pictures with extremely reduced recognizability after application of the transformation algorithm were excluded from the database – in these cases, all four different art pictures were removed.

Our final art.pics database includes three categories: animals (*n* = 90), objects (*n* = 413) and plants (*n* = 65) – each in four different art styles – adding up to a total of 2,332 art pictures (animals *n* = 360, objects *n* = 1,712 and plants *n* = 260).

### Deep Learning Algorithm (Art Filter)

On the basis of the concept by Gatys et al. (2015, 2016) a convolutional neural network (CNN) was used to turn the original pictures into stimuli with a specific art style. For this purpose, the pre-trained CNN named VGG-19 model (Simonyan and Zisserman, 2014) was used. This VGG-19 CNN was trained on 14 million images with 1,000 different categories. Thereby, the art style of one input image was applied onto another input image using an adapted version of the python code http://www.cvc.uab.es/people/joans/slides_tensorflow/tensorflow_html/neural_art.html with a TensorFlow implementation (©2007 Free Software Foundation). Options were set to 10% noise and 200 iterations.

### Image Characteristics

Image characteristics, i.e., “low-level” features, were derived using previously published scripts^4^ (Blechert et al., 2014) computed in Matlab R2019b (The Mathworks, Inc., Natick, MA, United States), and included the following: color (red, blue, and green), object size, intensity, normed intensity, complexity, normed complexity, mean and median power.

### Experimental Validation Study

In order to evaluate the created art.pics with regard to “high-level” features [i.e., liking, wanting, recognizability, arousal, and valence (the latter in a subsample, *n* = 1288)], we divided the database into twelve picture sets and asked 1,296 participants to rate the images. Pictures were randomly assigned to one of the twelve sets with the random()-function in python (version 2.7). Picture sets were presented in different orders to the raters for validation. The assignment of picture set to rater was randomized with the sample()-function in R (version 3.5). Validation of the pictures took place either at our institute (software programmed with Presentation 16.5) or through a survey created with Lime Survey (version 3)^5^. Prior to picture evaluation, we asked raters for demographics, namely sex, age, country of residence and occupation. We further assessed the raters’ art education and interest with the Vienna Art Interest and Art Knowledge Questionnaire [VAIAK (Specker et al., 2018)].

The institutional ethics board of the Medical Faculty of the University of Leipzig raised no concerns regarding the study protocol (228/18-ek) and all participants provided written informed consent.

Validation study was run in 1,296 participants mainly living in Germany (684f, age 30.2 ± 8.8 y.o.) who were recruited via online advertisement, local flyers in the institutes and via Prolific^6^ (720 participants). Some participants rated more than one picture set, resulting in a total of 1,391 ratings for each criterion. Five out of the 103 on-site participants conducted the rating via the English version of the presentation task. Normative rating data was collected for all stimuli for liking, wanting, recognizability, arousal and for valence. Ratings were acquired using the questions (1) liking (*How much do you like the picture?/Wie sehr mögen Sie das Bild?*) (2) wanting [*How much would you like to have it now* (e.g*., as a poster*)*?/Wie sehr hätten Sie das Bild jetzt gerne (bspw. als Poster*)?] (3) recognizability (*How recognizable is the object in the picture?/Wie erkennbar ist das Objekt auf dem Bild?*) (4) arousal (*How exciting do you find the picture?/Wie aufregend finden Sie das Bild?*) (5) valence (*How negative or positive is this picture for you?/Wie negativ oder positiv ist das Bild für Sie*?) on a Likert scale from 1 (not at all) to 8 (very much) or from −−−− to ++++ for valence.

Remuneration for artwork was realized by sending out (one of) the highest rated art images as a print or digital copy after the study. Participants were informed prior to the study, that rating of wanting was coupled to a real-life remuneration (= art print) of the most wanted image. With a real-life print as direct reward after the ratings that individuals were asked to take with them, we induced a situation where participants gained value for their ratings, i.e., a premium print, and thus their invested time and evaluation was rewarded by receiving their individually wanted picture.

Across previously published databases, ratings per picture varied between 14 (food-pics extended) to 108 (OASIS) participants (see Table 2). Guided by other databases, we estimated average ratings of 80 (M = 84.1, SD = 10.9) per picture to be sufficient for reliable ratings (statistical tests shown in *3. Results*).

**TABLE 2:** Characteristics of databases included in the creation of and the art.pics database itself.

	Food-pics	Food-pics extended	FRIDa	Open Affective Standardized Image Set (OASIS)	art.pics
Raters total (*n*)	*n* = 1988		*n* = 73	*n* = 900	*N* = 1296
Raters per image (*n*)	*n* = 48.8 (SD = 22.9)	*n* = 14–47 (M = 28.21 images, SD = 5.26)	*n* = 73	*n* = 101–108 (M = 103.25, SD = 2.77)	*N* = 64–127 (M = 84.1, SD = 10.4) subsample valence: *n* = 56–120 (M = 75.4, SD = 10.5)
Raters’ demographics	German-speaking and English-speaking cohort		39 females; 64 right handed	(420f, mean age 36.63 ± 11.91 and range from 18 to 74 years)	Mainly Germany (684f, age 30.2 ± 8.8 y.o.)
Stimuli (*n*)	568 food images 315 non-food images	Additional 328 food images	877 images (8 categories: natural-food, transformed-food, rotten-food, natural-non-food items, artificial food-related objects, artificial objects, animals, and scenes)	900 images	2,332 art images (animals, objects, plants; 8 art styles)
Reference	https://www.frontiersin.org/articles/10.3389/fpsyg.2014.00617/full#h3	https://www.frontiersin.org/articles/10.3389/fpsyg.2019.00307/full	https://www.frontiersin.org/articles/10.3389/fnhum.2013.00051/full#h3	https://link.springer.com/article/10.3758/s13428-016-0715-3	https://osf.io/BTWNQ/

### Data analysis and Statistics

Collected data (picture ratings and demographics) were fed into R (version 3.5.1) and statistics were conducted with the “BayesFactor” package^7^ using default settings, such as rscaleFixed *r* = 0.5 as a prior for Bayesian statistics. We used Bayesian linear modeling to compare picture ratings between categories (animals, objects, and plants), between art styles and to ensure that neither raters’ demographics nor picture ratings nor image characteristics differed between picture sets (1–12). In Bayesian statistics, the Bayes Factor (BF) is a measure of the strength of evidence in favor for one hypothesis among the other. A common interpretation is that if BF is larger than 3, the evidence favors the alternative hypothesis (H1), while it favors the Null hypothesis (H0) if BF < 1/3. As a full linear model we defined: (value of interest ∼ set + category + style) and divided this, respectively, by the null model leaving out the factor of interest. As Null hypothesis, we thus defined those models that did not include the factor of interest (i.e., category, age, sex, and image characteristics, etc.) as explaining variable. Values of interest were either the mean z-scored ratings or the image characteristics.

Z-transformation of the picture ratings was performed to render ratings of different participants comparable. Therefore all individual ratings (*x*) were z-scored for each participant and each criterion, respectively, ($z=\frac{x-\mu }{\sigma }$, μ = mean rating of all pictures per criterion per participant, σ = standard deviation of this mean rating).

To evaluate inter-rater reliability of the picture ratings, we calculated a reliability measure “R” using a resample methods according to Kurdi et al. (2017). Therefore, for each criterion, we split the ratings of each image randomly into two halves and took the mean of these halves in order to calculate the correlation between two “random raters” among pictures. We repeated this procedure 1,000 times per criterion to ensure the representability of the randomly generated halves. Additionally, we calculated intraclass correlation coefficients (ICCs) between raters of the same image subset – resulting in 12 ICCs which we averaged for each criterion. To do so, we used the ICC function in R from the “psych” package (version 2.0.9) which uses linear mixed models, reporting the ICC3 for a fixed set of participants who rate each image in the respective subset.

## Results

### Image Characteristics

**FIGURE 2:**
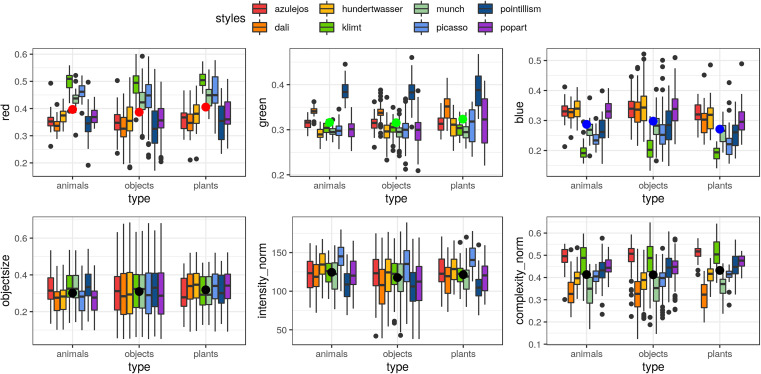
Overview of low-level image characteristics for the different categories (animals, objects and plants) and eight art styles, namely the ratio of red, green and blue as well as object size, normed intensity and normed complexity. (Boxplot showing mean as large dot).

All art.pics were characterized for low-level image features, namely the ratio of red, green and blue as well as object size, normed complexity and normed intensity. All values are available in the art-pics database. The Bayesian full/null model comparison of the low-level image characteristics did not show any significant differences across picture sets (see Table 3 for descriptives and statistics). Though, regarding the comparison across categories (animals, objects and plants; Figure 2) and art styles (“Azulejos,” “Dalí,” “Hundertwasser,” “Klimt,” “Munch,” “Picasso,” “Pointillism,” and “Popart”; Figure 2) all image characteristics were likely to be different (see Table 3).

**TABLE 3:** Descriptives and Bayes Factors of full/null linear model regarding the comparison of image characteristics across validation picture sets, categories (animals, objects and plants) and art styles (“Azulejos,” “Dalí,” “Hundertwasser,” “Klimt,” “Munch,” “Picasso,” “Pointillism,” and “Popart”).

Image characteristics	Mean + SD (range)	Full/Null linear model bayes factor ± error rate (image characteristics ∼ picture set)	Full/Null linear model bayes factor ± error rate (image characteristics ∼ category)	Full/Null linear model bayes factor ± error rate (image characteristics ∼ art style)
Red	0.39 ± 0.08 (0.17–0.60)	0.05 ± 1.8%	>4 × 10^4^ ± 2.41%	>2 × 10^363^ ± 2.11%
Green	0.32 ± 0.04 (0.21–0.47)	0.07 ± 2.17%	>1 × 10^3^ ± 2.38%	>9 × 10^504^ ± 2.62%
Blue	0.29 ± 0.07 (0.13–0.52)	0.08 ± 2.99%	>4 × 10^13^ ± 4.18%	>1 × 10^341^ ± 2.95%
object size	0.31 ± 0.12 (0.05–0.69)	0.16 ± 1.69%	0.03 ± 2.65%	1.1 ± 5.68%
Normed intensity	119.1 ± 26.2 (37.97–189.06)	0.16 ± 2.37%	>2 × 10^3^ ± 3.95%	>1 × 10^52^ ± 2.48%
Normed complexity	0.42 ± 0.09 (0.12–0.65)	0.14 ± 2.06%	48.95 ± 2.28%	> 3 × 10^289^ ± 2.49%

Low-level image characteristics showed wide ranges of red, green and blue color ratios (0.13–0.60) across categories and art styles (Figure 2). Overall, plants showed more red, green and less blue on average compared to animals and objects. Also more red colors were found consistently in Klimt, Picasso and Munch style pictures, more green colors in Pointillism, more blue in Azulejos, Dalí and Hundertwasser, and the least amount of blue in Klimt style pictures. Object size showed relatively high variance but no consistent patterns across categories or art styles. Normed intensity was highest for Picasso style images across all three categories, while on average being higher for animals compared to objects and plants. Normed complexity was slightly higher for plants, and consistently higher across all three categories for Azulejos and Klimt style pictures.

### Experimental Validation of the Database

**FIGURE 3:**
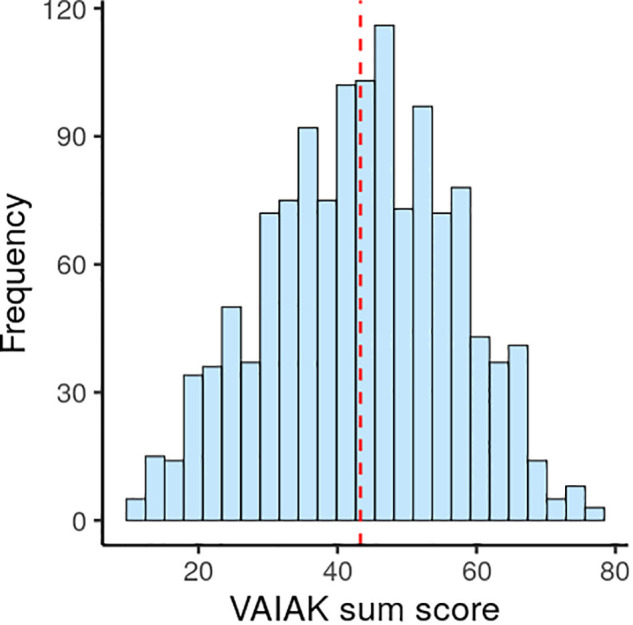
Distribution of VAIAK score in validation sample (*n* = 1296) (red line indicates mean). Possible score range is 11 to 77.

For rating higher-level characteristics along with every stimulus in the art.pics database, 1,296 individuals provided ratings (female = 684, male = 608, n.a. = 4; aged 30.2 ± 8.8 years, overall VAIAK score indicating artistic interest 43.3 ± 13.3 (maximum score possible = 77, Figure 3), country of residence: Germany = 759, Austria = 58, Switzerland = 18, others = 465; occupation: students = 590, full-time job = 425, part-time or irregular job = 181, retired = 11, unemployed = 89) (see Table 4). Bayesian linear modeling did not reveal any probable differences between raters’ demographics, source of recruitment or art knowledge and interest (measured with the VAIAK) across validation picture sets (see Table 4). Raw data of the raters and their demographics can be found in (Supplementary Table 2).

**TABLE 4:** Demographic characteristics of participants in the experimental validation.

Demographics		Linear model bayes factor ± error rate (Demographics ∼ Picture Set)
Sex	f = 684, m = 608, na = 4	0.34 ± 0%
Age	30.2 ± 8.8 y.o. (range: 16–70)	0.08 ± 0%
Country of residence	Germany = 759, Austria = 58, Switzerland = 18, and others = 465	0.07 ± 0%
Occupation	Students = 590, full-time job = 425, part-time or irregular job = 181, retired = 11, and unemployed = 89	0.06 ± 0%
Source of recruitment	on-site = 103, Prolific = 720, and other online recruitment strategies = 473	0.08 ± 0%
VAIAK score	43.3 ± 13.3 (range: 11–77) (possible score range 11–77)	0.001 ± 0%

### art.pics Ratings

**FIGURE 4:**
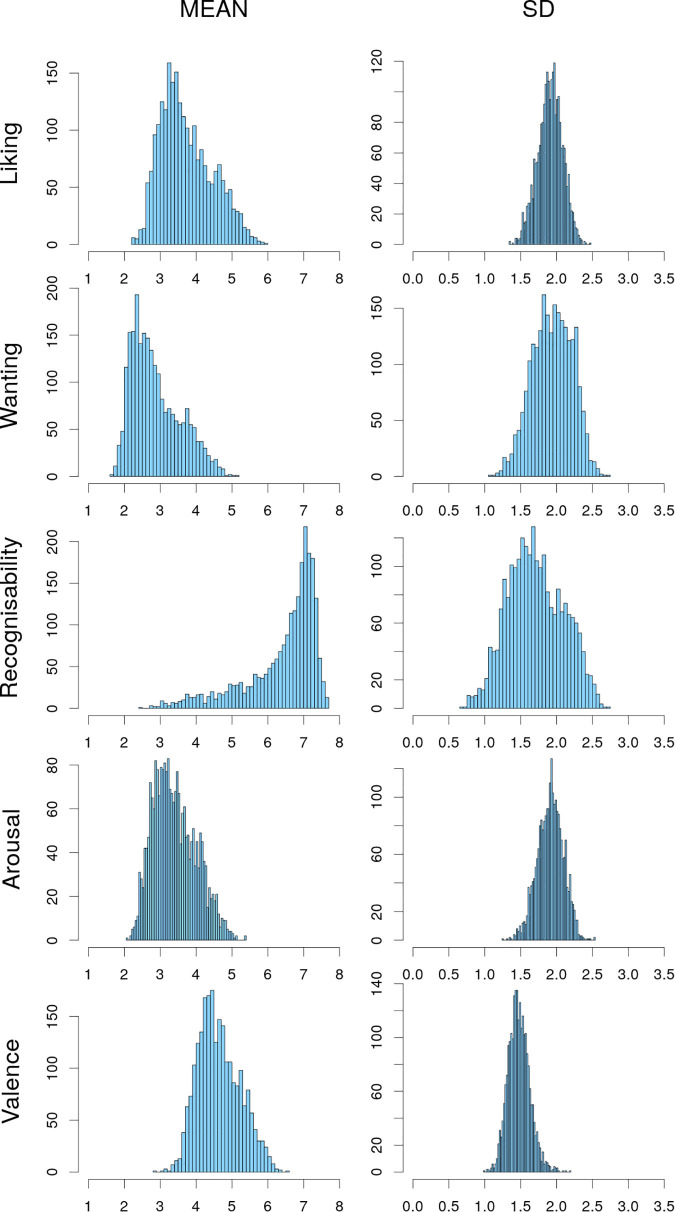
Univariate distributions of image-wise mean (left column) and standard deviations (right column) for liking, wanting, recognizability, arousal and valence ratings. All are normally distributed with a skewness between −1 and 1, except for the mean recognizability.

**FIGURE 5:**
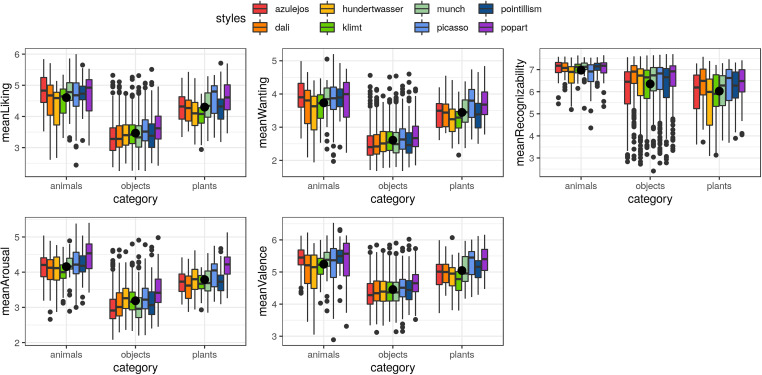
Overview of high-level image characteristics for the different art styles grouped by the three categories (animals, objects and plants), namely means across all participants for liking, wanting, recognizability, arousal and valence. (Boxplot showing mean as large dot).

High-level image characteristics were evaluated by the participants in the experimental validation study regarding five criteria, namely liking, wanting, recognizability, arousal and valence (in a subsample). The distribution of the imagewise means and standard deviations per criterion is shown in Figure 4. Mean values for liking were higher (3.7 ± 0.7, 2.3–6.0 points) compared to wanting (2.9 ± 0.7, 1.7–5.2 points) (see Figure 5 and Table 5 for descriptives and statistics). Recognizability on average was very high (6.4 ± 1.0, 2.4–7.7 points). Arousal on average was rather low (3.4 ± 0.6, 2.1–5.4 points), whereas valence was overall positive (4.6 ± 0.6, 2.9–6.5 points). Z-scored ratings are depicted in Supplementary Figures 1, 2. Raw data for every art.pic regarding categories, art styles, image characteristics and mean as well as z-scored ratings can be found in Supplementary Table 1.

**TABLE 5:** Descriptives of mean ratings per criterion and Bayes Factors of full/null linear model regarding the comparison of z-scored liking, wanting, recognizability, arousal and valence ratings across validation picture sets, categories (animals, objects, and plants), and art styles (“Azulejos,” “Dalí,” “Hundertwasser,” “Klimt,” “Munch,” “Picasso,” “Pointillism,” and “Popart”).

Criterion	Mean ± SD (range on 8-point scale)	Full/Null linear model bayes factor ± error rate (z-scored ratings ∼ picture set)	Full/Null linear model bayes factor ± error rate (z-scored ratings ∼ category)	Full/Null linear model bayes factor ± error rate (z-scored ratings ∼ art style)
Liking	3.7 ± 0.7 (2.3–6.0)	0.09 ± 2.93%	>3.7 × 10^257^ ± 3.24%	>4.8 × 10^8^ ± 3.06%
Wanting	2.9 ± 0.7 (1.7–5.2)	0.1 ± 1.56%	>1.3 × 10^293^ ± 1.80%	>1.7 × 10^6^ ± 1.79%
Recognizability	6.4 ± 1.0 (2.4–7.7)	0.04 ± 16.35%	>6.7 × 10^31^ ± 3.57%	4116.0 ± 4.84%
Arousal	3.4 ± 0.6 (2.1–5.4)	0.06 ± 2.65%	>1.0 × 10^272^ ± 2.77%	>1.2 × 10^41^ ± 2.74%
Valence	4.6 ± 0.6 (2.9–6.5)	0.07 ± 1.57%	>5.8 × 10^173^ ± 1.91%	>2.5 × 10^9^ ± 3.74%

The Bayesian full/null model comparison of the mean z-scored ratings for liking, wanting, recognizability, arousal and valence did not show any probable differences across validation picture sets. Regarding the comparison across categories (animals, objects and plants; Figure 5) and art styles (“Azulejos,” “Dalí,” “Hundertwasser,” “Klimt,” “Munch,” and “Picasso,” “Pointillism,” and “Popart”; Supplementary Figure 3) though, the mean z-scored ratings for all criteria were likely to be different (see Table 5).

High-level image characteristics compared between categories (see Figure 5 and Table 6) revealed that animals and plants were more liked and wanted by the raters than objects. Animals were also rated to be more recognizable than objects and plants. The same pattern as for the criteria liking and wanting applies as well to arousal and valence, indicating that animals and plants were perceived more emotionally and positively than objects.

**TABLE 6:** Descriptives of mean ratings per category (analogous to large dots in Figure 5). All z-scored criteria were likely to be different between categories (see Table 5).

Criterion	Categories Mean ± SD (range on 8-point scale)		
	Animals	Objects	Plants
Liking	4.60 ± 0.64 (2.44–6.00)	3.47 ± 0.54 (2.25–5.51)	4.30 ± 0.55 (2.94–5.71)
Wanting	3.73 ± 0.62 (1.94–5.20)	2.61 ± 0.48 (1.68–4.60)	3.44 ± 0.52 (2.16–4.84)
Recognizability	6.97 ± 0.46 (4.36–7.65)	6.35 ± 1.04 (2.41–7.70)	6.03 ± 1.05 (3.09–7.58)
Arousal	4.16 ± 0.46 (2.66–5.40)	3.19 ± 0.47 (2.08–4.98)	3.78 ± 0.42 (2.85–5.13)
Valence	5.24 ± 0.63 (2.89–6.53)	4.45 ± 0.46 (3.12–6.02)	5.05 ± 0.49 (3.72–6.16)

High-level image characteristics compared between art styles (see Supplementary Figure 3 and Table 7) showed slighter differences regarding all five criteria than the comparison between categories. The most consistent finding though is that images with the Popart style were rated highest in almost all five criteria – only Dalí style pictures were more recognizable to the raters.

**TABLE 7:** Descriptives of mean ratings per art style (analogous to large dots in Supplementary Figure 3). All z-scored criteria were likely to be different between art styles (see Table 5).

Criterion	Art styles mean ± SD (range on 8-point scale)							
	Azulejos	Dalí	Hundertwasser	Klimt	Munch	Picasso	Pointillism	Popart
Liking	3.68 ± 0.79 (2.37–5.84)	3.64 ± 0.73 2.25–5.71)	3.64 ± 0.62 (2.49–5.23)	3.69 ± 0.64 (2.26–5.34)	3.73 ± 0.76 (2.28–5.86)	3.83 ± 0.73 (2.25–6.00)	3.74 ± 0.73 (2.50–5.71)	3.92 ± 0.69 (2.42–5.70)
Wanting	2.84 ± 0.74 (1.71–4.99)	2.80 ± 0.67 (1.68–4.66)	2.79 ± 0.60 (1.77–4.42)	2.85 ± 0.61 (1.78–4.51)	2.88 ± 0.71 (1.80–5.05)	2.94 ± 0.71 (1.68–5.20)	2.87 ± 0.70 (1.79–4.93)	3.02 ± 0.68 (1.85–4.84)
Recognizability	6.18 ± 1.11 (2.83–7.58)	6.62 ± 0.88 (2.96–7.60)	6.29 ± 1.08 (2.72–7.60)	6.30 ± 1.04 (3.13–7.64)	6.46 ± 0.97 (2.41–7.65)	6.45 ± 1.02 (2.77–7.67)	6.40 ± 0.97 (2.78–7.70)	6.58 ± 0.91 (3.34–7.70)
Arousal	3.39 ± 0.47 (2.29–4.66)	3.33 ± 0.41 (2.32–4.88)	3.44 ± 0.42 (2.46–4.57)	3.39 ± 0.45 (2.14–4.65)	3.35 ± 0.47 (2.36–4.91)	3.45 ± 0.43 (2.38–4.75)	3.37 ± 0.42 (2.40–5.04)	3.49 ± 0.47 (2.45–5.40)
Valence	4.58 ± 0.63 (3.34–6.13)	4.57 ± 0.58 (3.12–6.23)	4.54 ± 0.54 (3.05–6.07)	4.58 ± 0.53 (3.45–6.00)	4.63 ± 0.60 (3.14–6.14)	4.69 ± 0.61 (2.89–6.53)	4.71 ± 0.59 (3.66–6.32)	4.83 ± 0.57 (3.31–6.16)

**FIGURE 6:**
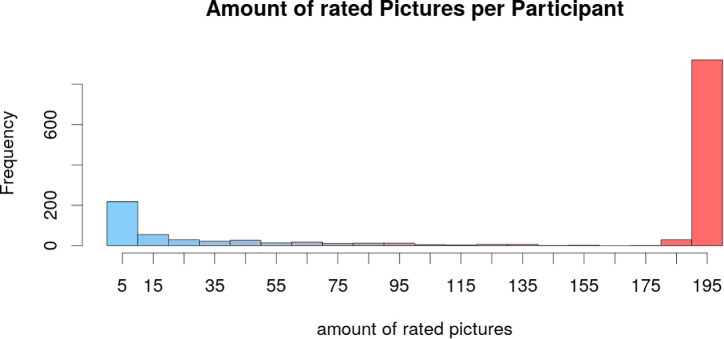
Distribution of the amount of pictures that were rated by each participant. Most raters completed the whole picture set that was assigned to them. Some raters only rated a very small amount of pictures which can be due to technical problems. Because of the anonymity of the ratings, this figure does not show when a participant rated more than one picture set ߝ this figure represents each picture set by itself.

Most raters completed the picture set they were assigned to, resulting in a maximum amount of 196 pictures per participant. In total, 921 participants rated more than 190 pictures (highest bar in Figure 6). Nevertheless, some participants only rated a very small amount of pictures which can be due to technical problems (amount of pictures between 1 and 10: *n* = 210, see Figure 6). Because of the anonymity of the ratings, we could not sum up the picture sets if participants rated more than one picture set.

**FIGURE 7:**
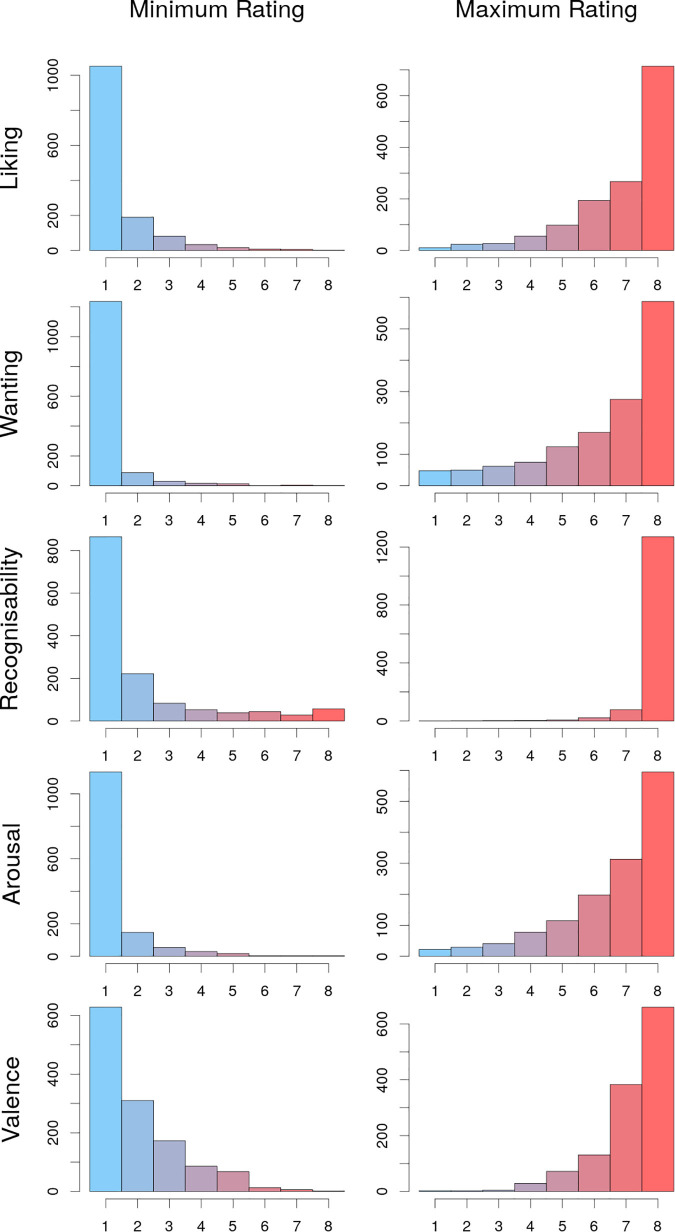
Distribution of minimal and maximal picture ratings of each rater regarding the different criteria. Most raters chose at least once a minimal rating of 1 and a maximal rating of 8 for all criteria.

In addition, most participants scored at least one picture with eight points for liking and wanting, respectively, (frequency of maximal ratings for liking: 6-points *n* = 194, 7-points *n* = 267, 8-points *n* = 714, and wanting: 6-points *n* = 170, 7-points *n* = 275, 8-points *n* = 587) (see Figure 7, right column, first and second plot from the top). Most raters also chose a 1-point rating for at least one picture (frequency of 1-point rating for liking: *n* = 1,033, and for wanting: *n* = 1213) (see Figure 7, left column, first and second plot from the top). Note that a certain proportion of participants (∼200) evaluated ≤10 images, most certainly due to technical issues, thus these might not have made use of the full rating scale. The usage of the full range of the 8-point Lickert scale can also be stated for the criteria recognizability, arousal and valence.

Considering inter-rater reliability, *R* values were considerably large for every criterion, whereas ICCs were small to moderate (see Table 8).

**TABLE 8:** Reliability measures R and ICC for the experimental validation study.

	R (resampling method)				ICC (for the 12 image sets)			
	Mean	SD	Min	Max	Mean	SD	Min	Max
Liking	0.84	0.004	0.83	0.86	0.18	0.03	0.10	0.22
Wanting	0.82	0.005	0.80	0.84	0.17	0.03	0.09	0.20
Recognizability	0.93	0.002	0.92	0.94	0.34	0.03	0.29	0.34
Arousal	0.77	0.006	0.75	0.80	0.13	0.01	0.10	0.15
Valence	0.84	0.004	0.83	0.86	0.13	0.02	0.10	0.16

## Discussion

We here provide a novel, large database of well-characterized, art stimuli with 2,332 items for use in experimental studies on secondary reward, (neuro-)esthetics and other social neuroscience fields. While comparable to other common visual stimulus material in terms of size, variable color distribution, complexity, intensity and valence, experimental validation by 1,296 raters in total, and 56–130 raters per image, showed that the art.pics elicit a broad and significantly different variation in subjective value ratings (i.e., liking, wanting) as well as in recognizability and arousal across different categories (animals, objects, and plants) and art styles. Individual ratings covering the full range from one to eight points, especially for liking and wanting, indicate that the art.pics stimuli elicit diverse subjective responses, resulting in a rich and extensive database of novel art stimuli. At the same time, the esthetic appeal of some art.pics were consistently rated higher than others, which is similar to other popular art picture compilations such as OASIS (Kurdi et al., 2017).

Besides subjective ratings, the variation in image characteristics, the three different categories and eight distinct art styles add up to the high diversity of this stimuli database, similar to common databases such as those on primary reward including food-pics (Blechert et al., 2019) and FRIDa (Foroni et al., 2013). Specifically, compared to food stimuli from the food-pics database, art.pics show similar mean ratings for arousal and valence, slightly lower recognizability and overall lower liking and wanting ratings. Similar arousal and valence ratings underpin that art.pics are a reasonable database to compare stimuli across scales (e.g., food and art images) in future studies. Lower recognizability of art.pics can be expected because of the morphing of the two images leading to reduced dimensionality. Lower wanting and liking ratings could be interpreted as a lower overall value attribution to secondary rewards (art compared to food as a primary reward). A direct comparison of our rating and wanting evaluation to previous databases is somewhat difficult given that liking and wanting ratings are not available in those databases. However, the OASIS database showed a somewhat similar distribution of valence (or pleasure) and beauty ratings (Kurdi et al., 2017; Brielmann and Pelli, 2019), further underlining the comparability and reliability of the art.pics.

### Image Characteristics and Ratings

We evaluated and compared all art stimuli of the database in terms of image characteristics and subjective value ratings between the 12 randomly generated validation sets, and between (1) categories (animals, objects and plants) and (2) art styles. Evaluation of the image characteristics of the 12 random sets showed that the picture sets for rating were very unlikely to be different in color distribution, complexity and intensity, rendering a bias due to these characteristics similarly unlikely. However, categories and art styles differed significantly in all image characteristics except for object size, which is most likely due to the fact that the original input art styles and categories differed as well in color, complexity, and intensity – which seems obvious to the naked eye, e.g., looking at the golden colors of the Klimt-style vs. the intense multi-colors of the Hundertwasser-style; or the predominantly red and green colors of plants vs. animals and objects.

Considering the experimental validation study, we were able to obtain ratings from a large sample of 1,296 participants living mainly in Germany (684f, age 30.2 ± 8.8 y.o.). Ensuring the generalizability of results, there were no differences in demographics or art knowledge between raters (measured with the VAIAK) across the 12 randomly assigned validation picture sets. Consequently, we can infer that random assignment of picture set to rater was successful and none of the sets were likely to be biased by sex, age, country of residence, occupation or art knowledge and interest. However, the ratings might not be generalized to Eastern or other populations as art styles and ratings are based on European cultural influence only (Bao et al., 2016).

The normal distribution of most of the mean ratings, with the exception of mean recognizability which was skewed to higher values, underscores the wide range of ratings and perceived esthetic value of the art.pics stimuli. Considering the ratings, there were no significant differences in any of the rating criteria across the 12 validation sets, so that we could exclude any biases introduced by the assignment of set to rater. However, we found higher liking, wanting and arousal values for animals and plants compared to objects. This seems intuitive as animals and plants rather evoke emotions than mere objects do, see Vessel et al. (2018) for further discussion.

Inter-rater reliability measures largely differed between the resampling method and the ICC method (delta = ∼0.65), which seems insightful given that averaging the split halves-ratings in the resampling method should have substantially reduced/softened differences between the underlying actual ratings. Thus, the correlation R values between the “two” resampled raters resulted in high scores (from 0.77 to 0.93 for all criteria), which is similar to the reliability scores for the ratings of the OASIS database (Kurdi et al., 2017). In contrast, ICC values of the individual art.pics ratings resulted in mean ICCs ranging from 0.13 to 0.34, suggesting that the correlations between on average about 107 participants per image subset was rather poor. This does not necessarily mean that the quality of the ratings was low but rather emphasizes that subjective evaluation differed greatly over almost 200 images (per subset) and that our participants can represent a larger population.

It has also been shown that the color composition has a high impact on perceived beauty of paintings irrespective of naturalistic representation for professional painters (Nascimento et al., 2017) which could explain the differences in subjective ratings of categories and art styles that differ in color distribution. Recognizability for plants was lowest which might be due to larger disfigurement during transformation of the on average more complex images compared to animals and objects and which might impact generalizability in certain scenarios. To obtain well-balanced stimuli sets in terms of generalizability, we recommend to exclude rather abstract pictures based on mean recognizability score <3 (or mean z-scored recognizability <−2) listed in Supplementary Table 1. Valence values collected in a subsample of the raters did not differ neither between categories nor art styles. Regarding the comparison of ratings across the eight art styles, we found significant differences for all criteria. For instance, Popart art.pics were rated on average highest in liking, wanting, arousal and valence whereas Dalí art.pics were on average most recognizable (see Table 7) - albeit with large differences between categories (see Supplementary Figure 3). This emphasizes the diversity of the chosen categories and art styles. In sum, even though we might not all agree on whether an artwork can be considered art, the provided subjective value ratings of the art.pics distribute over a large range fitting “each to their own,” showing that the database is a valuable stimuli collection to suit subjective tastes of art.

### Further Outlook and Applications of art.pics Database

The art.pics stimuli may be used in multiple experimental settings. One application could be task-dependent fMRI paradigms relying on visual cues requiring control stimuli to contrast scales of interest in the statistical analysis pipeline, e.g., *faces vs. houses* or *arousal vs. neutral*. Now, our well-characterized art stimuli add novel easy-to-use material of validated images to use in e.g., task-related fMRI on art experience, social exchange and secondary reward evaluation. In food-related neuroimaging common practice is to contrast *high- vs. low-caloric food* items [reviewed in Smeets et al. (2019)]. However, this contrast cannot inform about non-food rewards, e.g., when aiming to compare value signals across reward domains such as food items versus art paintings (Lebreton et al., 2015). In addition, food-related neuroimaging often suffers from a lack of specificity, e.g., when another reward domain contrast is not implemented in the task. This can now be improved using our artistic images. Further, the targeted brain activity in each experimental setting needs to be considered (viewing, executive control, mental imagery, liking, wanting, repulsion, and feedback) for implications in the study design, such as actual reward remuneration online or offline to the experiment. Rewards such as food, water and money are manageable to remunerate to subjects in the laboratory context; however, esthetics, sexual, and social cues need more creative solutions to be rewarded directly after the experiment, if at all possible. Therefore, we propose to print the art.pics on paper to ensure a realistic remuneration of artistic images and to use the database as a new (remunerable) contrast condition for food-related neuroimaging and other fields of research.

We hereby provide useful and high-quality stimuli that will enable more diverse experimental designs in the context of valuation paradigms in psychological and neuroimaging studies. Possible applications for art.pics images could be stimuli in behavioral as well as neuroimaging studies in the context of art-related research questions or printed on paper as reward remuneration for subjects after study participation. Well-characterized and widely used databases such as art.pics will help to increase comparability across study results and to promote more research on the understanding of art processing as such – but especially as an important control condition in fMRI studies.

## Terms of Use

We provide the database free of charge under a creative commons license on the basis of a license agreement completed by the supervisor/PI/Professor of a work group using the pictures. They would be responsible for the use of the art.pics in the work group in keeping with the license agreement. This is relevant for issues such as storage on shared network spaces, instruction of present and incoming students regarding license content. Data is stored on OSF (https://osf.io/BTWNQ/) and freely available. To obtain rights of use, please fill out the license agreement form found in OSF and send to AW, witte@cbs.mpg.de.

## Data Availability Statement

The datasets presented in this study can be found in the article under “Terms of Use” and in the Supplementary Material.

## Ethics Statement

The studies involving human participants were reviewed and approved by Medical Faculty of the University Leipzig. The participants provided their written informed consent to participate in this study.

## Author Contributions

RT, LD, EM, and AW: study conception/design and manuscript. RT, LD, and EM: data collection. RT and EM: data analysis. All authors contributed to the article and approved the submitted version.

## Conflict of Interest

The authors declare that the research was conducted in the absence of any commercial or financial relationships that could be construed as a potential conflict of interest.

## Footnotes

1 http://library.princeton.edu/resource/3585

2 http://www.eat.sbg.ac.at/resources/food-pics

3 https://foodcast.sissa.it/neuroscience/

4 www.food-pics.sbg.ac.at

5 https://survey3.gwdg.de/

6 https://www.prolific.co/

7 https://cran.r-project.org

## Supplementary Material

Supplementary Figure 1 Overview of mean z-scored ratings for the different categories (animals, objects and plants) regarding liking, wanting, recognizability, arousal and valence.

Supplementary Figure 2 Overview of mean z-scored ratings for the different art styles regarding liking, wanting, recognizability, arousal and valence.

Supplementary Figure 3 Overview of high-level image characteristics for the different categories (animals, objects, and plants)grouped by the eight art styles, namely means across all participants for liking, wanting, recognizability, arousal, and valence. (Boxplot showing mean as large dot).

Supplementary Table 1 Overview of all art.pics including descriptions, image characteristics, mean and z-scored ratings.

Supplementary Table 2 Overview of all participants in experimental validation study including demographics, assigned picture set and site of participation.
