# Supplementary material for: art.pics Database: An Open Access Database for Art Stimuli for Experimental Research
Source: Front Psychol. 2020 Dec 16;11:576580. doi: 10.3389/fpsyg.2020.576580 (PMC7772247; doi:10.3389/fpsyg.2020.576580)
Supplement: Supplementary Figure 1 — Overview of mean z-scored ratings for the different categories (animals, objects and plants) regarding liking, wanting, recognizability, arousal and valence. [file Presentation_1.pdf]

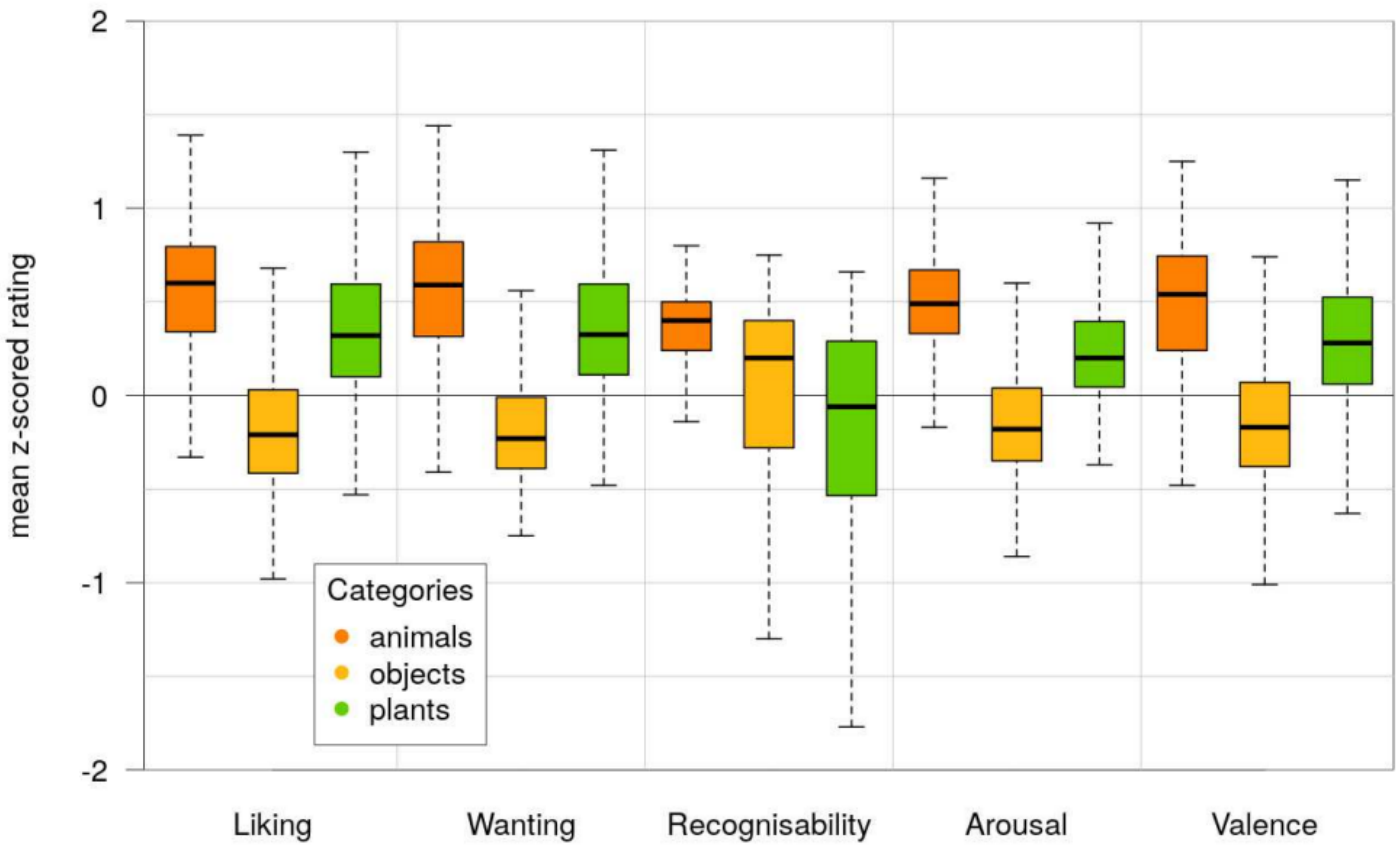

Supplementary Figure 1

Overview of mean z-scored ratings for the different categories (animals, objects, and plants) regarding liking, wanting, recognizability, arousal and valence.

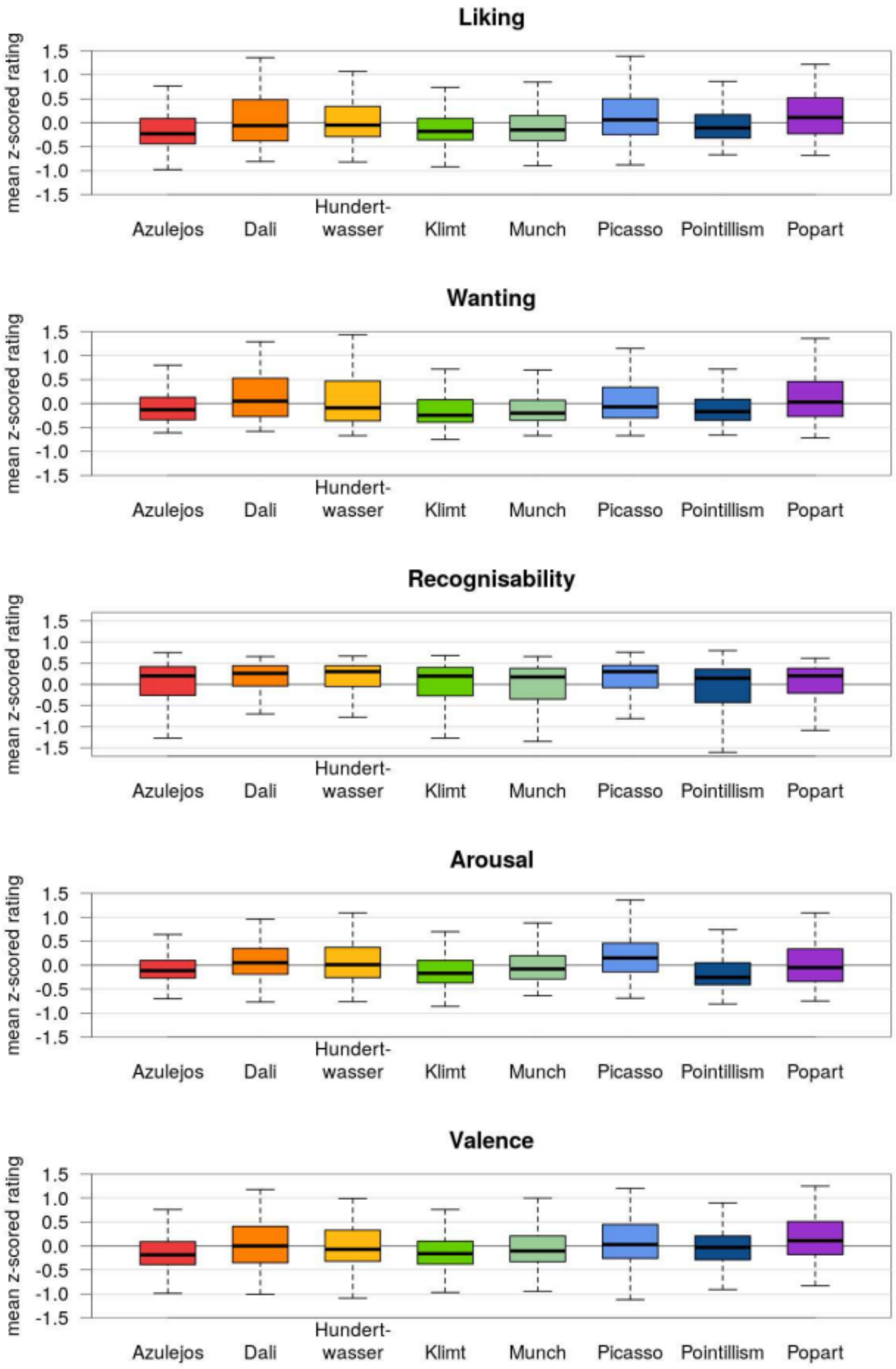

Supplementary Figure 2 :

Overview of mean z-scored ratings for the different art styles regarding liking, wanting, recognizability, arousal and valence.

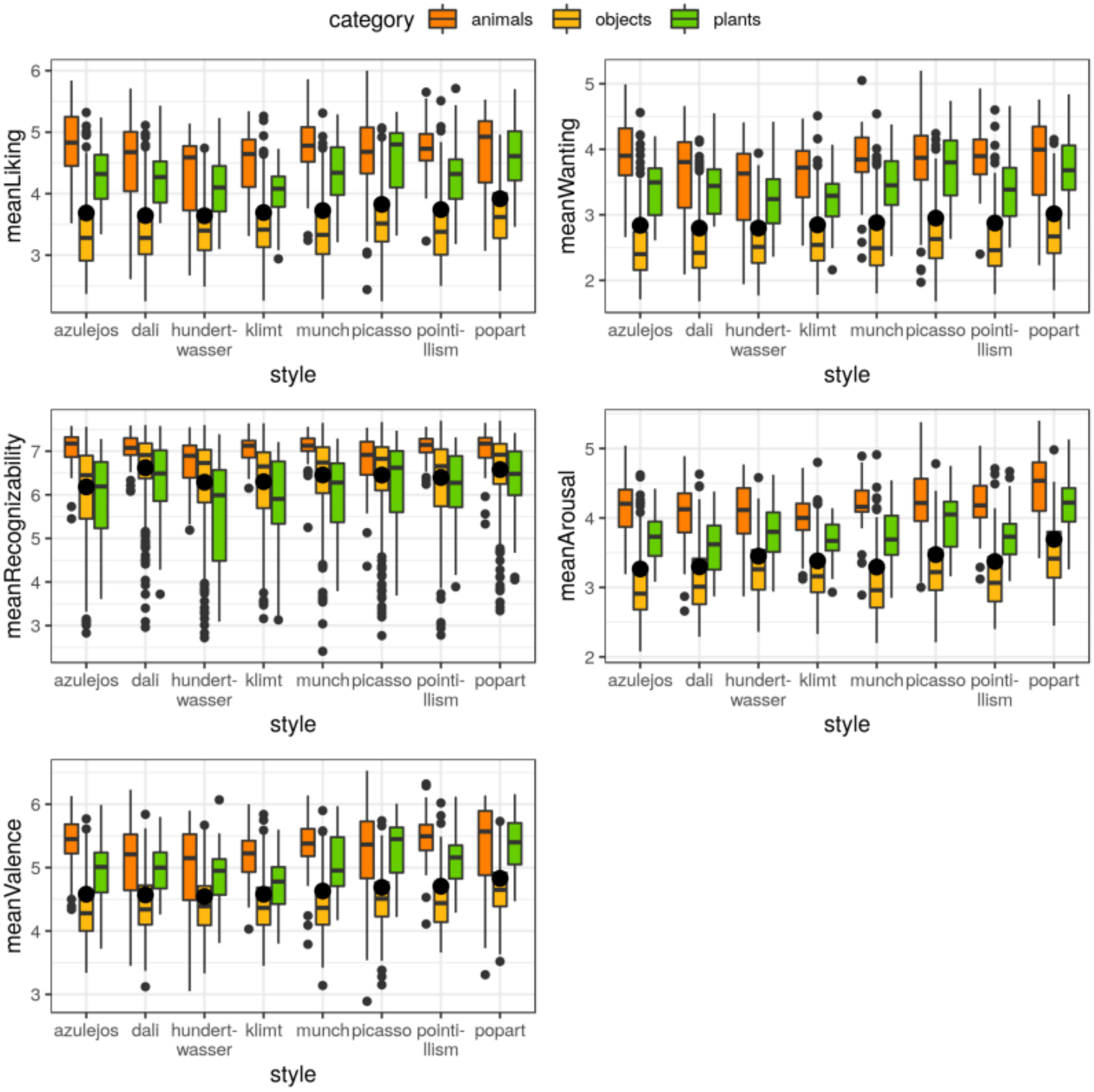

Supplementary Figure 3:

Overview of high-level image characteristics for the different categories (animals, objects, and plants) grouped by the eight art styles, namely means across all participants for liking, wanting, recognizability, arousal, and valence. (Boxplot showing mean as large dot).
